# Association between Body Mass Index and depression: the "fat and jolly" hypothesis for adolescents girls

**DOI:** 10.1186/1471-2458-11-649

**Published:** 2011-08-16

**Authors:** Anne Revah-Levy, Mario Speranza, Caroline Barry, Christine Hassler, Isabelle Gasquet, Marie-Rose Moro, Bruno Falissard

**Affiliations:** 1 INSERM, U-669 PSIGIAM, Paris, F-75679, France; 2 Univ. Paris-Sud, Univ. Paris-Descartes, Paris, F-75005, France; 3 Centre de Soins Psychothérapeutiques de Transition pour Adolescents, Hôpital d'Argenteuil, F-95107, Argenteuil, France; 4 Centre Hospitalier de Versailles. Service de Pédopsychiatrie. Le Chesnay, France; 5 AP-HP, Direction de la Politique médicale, Paris F-75004, France; 6 AP-HP, Hôpital Cochin, Maison de Solenn, Paris, F-75014, France; 7 AP-HP, Hôpital Paul Brousse, Département de Santé Publique, Villejuif, F-94804, France

## Abstract

**Background:**

Results concerning the association between Body Mass Index (BMI) and depression in adolescence are conflicting, some describing a linear association (increase in BMI with level of depression), some a U-shaped association (both underweight and obesity are associated with high levels of depression), and they mostly concern small samples. The purpose of this study was to describe the association between BMI and depression in a large representative sample of French adolescents.

**Methods:**

The association between BMI and depression, measured on the Adolescent Depression Rating Scale (ADRS), was tested in a French national representative sample of 39542 adolescents aged 17. Self-report data is derived from the 2008 ESCAPAD study, an epidemiological study based on a questionnaire focused on health and drug consumption. We used spline function analysis to describe the association between BMI and depression.

**Results:**

The association between BMI and depression is significant (*p *< 0.001) and non-linear for both genders, with no effect of parental working and marital status. For boys, there is U-shaped association. For girls the shape of the association is complex and shows inverted convexity for high levels of BMI. The spline shows higher scores for depression among overweight girls than among obese girls.

**Conclusion:**

There is evidence for a gender difference in the association between BMI and depression in adolescents, supporting the need to study boys and girls separately. Overweight adolescent girls are more likely to be depressed than obese adolescent girls, giving support for "fat and jolly" hypothesis not only among older women but also among adolescent girls.

## Background

Adolescence is a critical period, whether for weight problems or for depression. Obesity and depressive disorders in adolescence in particular are risk factors for chronic pathologies in adulthood, the medical and economic consequences of which are central public health issues [1-4]. Numerous covariates of the association between variation of weight and depression have been described, especially in adolescence, such as age, gender, socioeconomic status of the parents, family structure, level of education, race/ethnicity, body image, [5-9]. A multifaceted approach to explaining child and adolescent variations in weight has suggested that the home environment and socioeconomic status are important considerations [10,6].

While only longitudinal studies are likely to demonstrate the complex underlying mechanisms linking variation in weight and depression, such as a bidirectional association, with potential moderators such as physical activity, eating patterns, or depression severity [11-13], cross-sectional studies in a large-scale community sample do contribute to describing the characteristics of the association between weight and depression [14]. To date, all kinds of associations have been described at different ages: a positive association between depression and obesity (more severe depression is associated with greater obesity) [15-18], a negative association (more severe depression is associated with a lesser degree of obesity)[19], no association [20,21]. We found few studies in which the existence of a U-curve was tested [22-24]. According to Wardle, the case for a relationship between adolescent obesity and depression is not yet proven. The heterogeneity of results in this area could be related to different factors. Firstly adolescence is a period of bodily change, with a particular impact of weight change, probably more complex than in adults. Secondly, covariables envisaged differ from one study to another. And finally heterogeneity in results may also arise from the different methods used to measure depression [4,19].

Indeed, the variability of criteria to define categories of weight and depression from one study to another render interpretation of results rather difficult. There is also a problem with the use of depression measures that contain an item or items concerning appetite or weight, which could bias results.

To date, there has been no study on this question in any European country on a large sample of adolescents aged 17 and representative of that population. The aim of the present study, in the largest-ever representative sample of French adolescents aged 17 years-old, was i) to determine the prevalence of overweight and obesity, ii) to determine the prevalence of depression, using a validated tool, the Adolescent Depression Rating Scale (ADRS) [25], which contains no item on weight, appetite, or body image, and iii) to investigate the association between BMI and depression controlling for working and marital status of the parents.

## Method

### Participant selection

Participants were recruited in metropolitan France (i.e. excluding overseas territories) between March 15^th ^and March 31^st ^2008 during the National Defense Preparation Day (*Journée d'Appel de Préparation à la Défense *- JAPD). The JAPD is a civic and military information session that is required of all adolescents aged 17, and mandatory to sit public examinations (e.g., driving license, university exams). All French adolescents aged 17 years are called to participate in these national days in one of the 250 centers in France. In all, 764 000 adolescents aged 17 were living in metropolitan France in 2008. Among these, 44 733 (5.9%) subjects aged 17 years were invited to participate in the Survey on Health and Behavior, called ESCAPAD [26], a cross-sectional survey conducted by the French Monitoring Center for Drugs and Drug Addiction (*Observatoire Français des Drogues et des Toxicomanies*- OFDT), and administered during JAPD days in collaboration with the National Service Bureau of the Army. The sample finally included in this study comprised 39 542 French subjects living in metropolitan France (N = 19,658 girls and 19,884 boys). This represents 5.2% of adolescents aged 17 years living in metropolitan France (5.3% for girls and 5.1% for boys). The survey obtained the public statistics general interest seal from the *Comité National de l'Information Statistique *- CNIS, as well as the approval of ethics committee (Commission Nationale d'Information et Liberté).

### Measures of BMI and depression

From the ESCAPAD questionnaire, we used the data available about weight, height, and ADRS scores.

BMI was calculated as weight in kilograms divided by height in meters squared, based on self-reported heights and weights. According to WHO guidelines [27], adolescents were classified as underweight (BMI < = 17.0 kg/m^2^), normal weight (BMI of > 17.0-24.0 kg/m^2^), overweight (BMI of > 24-29.0 kg/m^2^), or obese (BMI > 29.0 kg/m^2^) and the proportions of subjects in these categories were computed separately for boys and girls.

Depression was measure using the ADRS, specifically developed to measure the intensity of depression among adolescents. This measure was previously validated on young people aged 12 to 20 and published with the official cut-off [25]. It is a 10-item self-administered questionnaire with yes/no responses concerning the two weeks preceding completion. The sum of item scores provides a score that divides the population into three distinct groups: score 0 to 2 "not depressed", 3 to 5 "sub-threshold depression", and 6 or more "depressed". The cutoff of 6 was chosen because it provides maximum sensitivity and specificity in screening for major depressive state according to DSM-IV with clinically relevant intensity, which corresponds to a CGI score (Clinical Global Impression) of 5 or more (i.e. markedly ill or more). The corresponding subjects are therefore termed "depressed" here. The group with a score of 3-5 is the sub-threshold group as defined in [25]. We used categorical data only for descriptive analyses but we used continuous data to investigate associations between ADRS and BMI.

Parental working and marital status were based on adolescent reports.

### Statistical analysis

Data are presented as means and standard deviation or percentages. Descriptive statistics were compared using Student t-test or the chi-square test.

The association between BMI and ADRS was studied using a thin-plate penalized regression spline model. This statistical method is based on the estimation of a general additive model with a penalized regression spline. This model is similar to a linear regression model with a dependant variable (here the ADRS score as a continuous numerical variable) and an explanatory variable (here BMI). But in contrast to linear regression, the explanatory variable BMI is not analysed *per se*, it is optimised using a piecewise cubic spline function. This cubic spline function aims to find the best-fitting curve representing the link between the dependent and independent variables, using cubic polynomials on a succession of consecutive intervals [28]. To avoid over-parameterization of the fitted model, a constraint is added to the algorithm, giving a so-called penalized regression spline model. The R software and the "mgcv" package were used for the analyses [29]. To statistically test the "inversion of convexity" observed in the regression spline between BMI and ADRS scores, a polynomial regression was estimated. More precisely, for a BMI between 25 and 35, ADRS was regressed against BMI expressed with 3 orthogonal polynomials of degrees respectively equal to 1, 2 and 3. The hypothesis was that the component of degree 3 should be statistically significant. A probability level of P < 0.05 was used to indicate statistical significance. The association was controlled for parental working and marital status in the linear part of the regression model, which is therefore semi-parametric.

## Results

Of the total sample (n = 39542), 49.7% were girls and 50.3% were boys. 92.8% of the parents had a professional activity and 71.2% were living together.

Our results show that the distribution of the sample across BMI categories differed significantly between boys and girls (p < 0.001). The prevalence of « thin » subjects was higher among girls than boys (4.0% *versus *2.2%), the prevalence of overweight was higher among boys than girls (12.9% *versus *8.7%) while the difference in the prevalence of obesity was not statistically significant (2.1% *versus *1.8%) (Table 1). Prevalence of overweight and obesity was higher when the parents were not working (p < 0.001) for both genders. We found a significant difference according to parental matrimonial status for the girls (p = 0.044) but not for the boys (Table 2).

**Table 1:** Demography, anthropometry and depression

	Boys **n = **19884	Girls **n = **19658	Total **n = **39542
**Demography**			
**Age **years (sd)	17.1 (0.29)	17.1 (0.27)	17.1 (0.28)
**Parents working **%			
yes	93.2	92.4	92.8
no	6.8	7.6	7.2
**Parents together **%			
yes	71.1	71.2	71.2
no (1)	28.8	28.7	28.8
**Anthropometry**			
**Weight **Kg (sd)	67.6 (10.3)	56.3 (8.5)	62.0 (11.0)
**Height **cm (sd)	177.3 (7.0)	164.9 (6.3)	171.1 (9.1)
**BMI **mean (sd)	21.5 (2.9)	20.7 (3.0)	21.1 (3.0)
**BMI category, %**			
< = 17.0	2.2	4.0	3.1
> 17.0-24.0	82.8	85.5	84.1
> 24.0-29.0	12.9	8.7	10.8
> 29	2.1	1.8	1.9
**Depression**			
**ADRS mean (sd)**	1.3 (1.9)	2.2 (2.3)	1.7 (2.1)
**ADRS score %**			
0-2	80.2	64.0	72.0
3-5	15.2	25.5	20.0
> = 6	4.5	10.4	7.5

Source: ESCAPAD 2008, OFDT

(1) This category includes young people whose parents are divorced or separated for other reasons, and also those who have lost one or both parents

Notes: sd = standard deviation;

**Table 2:** Parental status and BMI

	BMI category Boys				BMI category Girls			
	
	Under weight	Normal	Over weight	Obese	Under weight	Normal	Over weight	Obese
	< = 17	> 17-24	> 24-29	> 29	< = 17	> 17-24	> 24-29	> 29
**Parents working**								
yes	2.2	83.3	12.6	1.9	4.1	86.0	8.3	1.6
No	2.1	79.4	15.6	3.0	3.1	80.0	12.7	4.3
**Parents together**								
Yes	2.4	82.9	12.7	2.1	4.2	85.7	8.3	1.8
no (2)	1.9	83.1	13.0	2.1	3.4	85.2	9.5	1.9

Source: ESCAPAD 2008, OFDT

(2) This category includes young people whose parents are divorced or separated for other reasons, and those who have lost one or both parents.

Concerning depression, the mean score on the ADRS was significantly higher for girls compared to boys (p < 0.001). The proportion of depressed girls (ADRS score ≥ 6) (10.4%) was greater than that for boys (4.5%) (p < 0.001, Table 1). The prevalence of depression was significantly higher when parents were not working (p < 0.001), and when parents were separated (p < 0.001) for the two depression groups, sub-threshold and depressed for boys and for girls (Table 3).

**Table 3:** Parental status and depression

	ADRS score Boys			ADRS score Girls		
	
	Not depressed 0-2	Sub-threshold depressed 3-5	Depressed ≥ 6	Not depressed 0-2	Sub-threshold depressed 3-5	Depressed ≥ 6
**Parents working**						
yes	80.7	15.0	4.3	64.9	25.2	10.0
no	76.7	17.4	5.9	57.4	29.1	13.6
**Parents together**						
yes	81.3	14.6	4.2	65.8	24.5	9.7
no (2)	77.6	17.1	5.3	60.4	27.8	11.8

Source: ESCAPAD 2008, OFDT

(2) This category includes young people whose parents are divorced or separated for other reasons, and those who have lost one or both parents.

**Figure 1:**
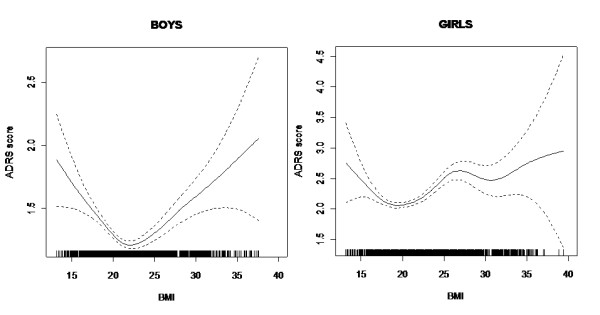
**Figure 1A and B: Association between BMI and depression (ADRS score) for boys and for girls**.

Depression and BMI were significantly associated in both the total sample, and in boys and girls separately (P < 0.0001 in all three samples). In addition, the association was not linear and the curve presented a different shape for boys and girls. (Figure 1A and 1B). Among boys, the U-shaped curve demonstrated that both obesity and underweight were associated with an increased level of depression (Figure 1A). Among girls, the curve showed a first part of the curve describing a U shape and then inverted convexity for higher BMI levels (Figure 1B) indicating that overweight adolescent girls were more likely to be depressed than obese adolescent girls.

In order to test whether this inversion of convexity observed in girls with BMI between 25 and 35 was statistically significant, we used a linear regression model with orthogonal polynomials. More precisely, for girls with BMI between 25 and 35, the ADRS score was regressed against BMI expressed with a linear quadratic and cubic component. The inversion of convexity corresponds, from a statistical point of view, to the cubic component, which was statistically significant here with p = 0.003 (the linear and quadratic components are not significant at the 5% level). The association did not alter when working and marital status were controlled for.

## Discussion

Obesity and depressive disorders are central public health issues and the relationship between depression and weight remains unclear [1,2].

Our study shows that irrespective of gender, the prevalence of overweight and obesity at 17 years of age is respectively 10.8% and 1.9%. Figures in France at age 14 are 7% for overweight and 4% for obesity [30,31]. According to gender, the present results show a prevalence of overweight (12.9% for boys and 8.7% for girls) that is comparable to that found in the international literature, ranging from 5.2% to 28.9% for boys and from 8.1% to 31.0% for girls [19]. As in the literature, we found differences in prevalence according to parental professional status and parental marital status [6,30,31].

The prevalence of depression in the present study on the basis of the ADRS measure specific to adolescents was 4.5% among boys and 10.4% among girls. In the literature, the data on prevalence of depression varies, in particular according to the type of measure used (measures of intensity, or DSM-IV-based) as well as according to whether or not gender differences are sought. In France, using criteria for depression according to a standardized assessment (CIDI short form), the prevalence of depression for the age of 15 to 24 years old as been reported to range from 6.3% for boys, to 11.2% for girls [32]. A review of recent epidemiological studies showed prevalence rates ranging from 1.8% to 5.9% in the United States [33-35]. For the years 2005-2006, the Center for Disease Control found a prevalence of depression between 4% and 6.4% for ages 12 to 17, using the PHQ-9, but without testing for gender differences [36]. Many epidemiological and clinical studies have shown that girls have typically been found to display higher levels of depressive symptoms than boys [37,38]. This has been attributed to genetics, increased prevalence of anxiety disorders in females, biological changes associated with puberty, cognitive predisposition and socio-cultural factors [39].

The most important result of our study concerns the non-linear and significant association between depression and BMI, with no effect of parental working and marital status. In addition, the shape of the association is different for boys and girls. We have a U- shaped association for boys, which shows that boys that are too thin or too fat have higher levels of depression. For girls, we have a beginning of a U shape, and then an inversely convex curve, which means, first, that depression levels in underweight girls are higher than in normal weight girls, and second, that depression levels are higher in overweight adolescent girls than in obese girls. This can be related to the "fat and jolly" hypothesis, which has been discussed for older women [40-42]. Our results recall the study by Wardle et al [19], who found, in a small sample of adolescents, that regardless of moderators reports of depressive symptoms were more frequent in overweight than in obese adolescent girls of 14, and who found no difference for depressive symptoms between normal weight and obese girls. Using splines, Cortese [5] in a small sample found curves of different shape for boys and a U-shaped curve for girls aged 11 to 14, showing that underweight girls had an equivalent level of depression to that of obese girls, this not being true for the boys. While some previous studies have explored the association between obesity and depression without questioning the gender difference [23,43,44], according to the recent meta-analysis of community-based studies by de Wit et al [14] there is some support for the hypothesis that there is a difference between males and females in the association.

The "fat and jolly" hypothesis, which we suggest for the adolescent girls of 17 years old, is generally thought to be related to hormone and neuro-endocrine issues, but it could be envisaged as a possible dynamic in adolescent girls. It can indeed be thought that the difficult struggle against excess weight could let up when the subject reaches the obesity stage, as if, once they give up the struggle, they may feel less depressed. In all events, obesity, despite creating negative social experiences in adolescence, does not in itself cause depression. The effect of moderators needs to be explored in the association between weight and depression, and in particular the complex association between weight and the perception of weight, as a risk factor of depression [45,46].

Several limitations of the study must be considered. BMI was calculated from self-report, with the attendant risk of under-estimation of weight. The degree of divergence between subjective and objective BMI has been described for different ages, different countries and according to gender [47]. However, most studies use self-report data. The underestimation tends to be greater among obese adolescents, so that the prevalence in our study may well be underestimated. Secondly, as our study is cross-sectional, we cannot demonstrate the mechanisms of the association found. Only longitudinal studies are able to explore the different effects of the potential moderators of the association, and investigate the possible predictive relationship between overweight and depression. Indeed, longitudinal studies can provide evidence for depression predicting future obesity or weight gain or the reverse. Published research to date has shown a mixed picture [11,48,49]. Thirdly, we have no data available about race or ethnicity because no studies in France are allowed to ask questions about theses major factors in public health. Moreover, the present data only concerns French nationals from metropolitan France (i.e. excluding overseas territories). However the sample nevertheless represents 5.2% of the French metropolitan population aged 17, and is representative of it.

Overall, since the results are derived in part from the visual examination of regression splines, they should be considered basically as exploratory, and therefore as requiring replication. Despite these limitations, this study provides valuable information on account of the exceptionally large size of the sample. To our knowledge, this is the very first survey worldwide on such a large representative sample of a total population of adolescents. And another important point is that while most studies use depression measures comprising items concerning the body, food, or weight, which are confounders in the exploration of the links between depression and weight, in the present study we used the ADRS measure of depression, which is the only measure that does not contain these confounders.

## Conclusion

These results point to the importance of distinguishing between girls and boys when we investigate the nature of the association between depression and the four different BMI categories. It also shows that overweight adolescent girls are at higher risk of depression than the obese adolescent girls.

## Competing interests

The authors declare that they have no competing interests.

## Authors' contributions

ARL wrote the first draft and the revisions of the manuscript. MS, CH and CB, BF conducted the statistical analyses. MS, IG, BF, MRM critically read each draft and contributed to the further drafting and revisions of the manuscript. All authors read and approved the final manuscript.

## Pre-publication history

The pre-publication history for this paper can be accessed here:

http://www.biomedcentral.com/1471-2458/11/649/prepub
